# Mechanisms of action behind the protective effects of proactive esophageal cooling during radiofrequency catheter ablation in the left atrium

**DOI:** 10.1016/j.hroo.2024.05.002

**Published:** 2024-05-15

**Authors:** Samuel Omotoye, Matthew J. Singleton, Jason Zagrodzky, Bradley Clark, Dinesh Sharma, Mark D. Metzl, Mark M. Gallagher, Dirk Grosse Meininghaus, Lisa Leung, Jalaj Garg, Nikhil Warrier, Ambrose Panico, Kamala Tamirisa, Javier Sanchez, Steven Mickelsen, Mayank Sardana, Dipak Shah, Charles Athill, Jamal Hayat, Rogelio Silva, Audra T. Clark, Maria Gray, Benjamin Levi, Erik Kulstad, Steven Girouard, Will Zagrodzky, Marcela Mercado Montoya, Tatiana Gomez Bustamante, Enrique Berjano, Ana González-Suárez, James Daniels

**Affiliations:** 1 Cleveland Clinic, Cleveland, Ohio; 2 WellSpan York Hospital, York, Pennsylvania; 3 St. David’s Medical Center, Texas Cardiac Arrhythmia Institute, Austin, Texas; 4 Ascension St. Vincent, Indianapolis, Indiana; 5 NCH Rooney Heart Institute, Naples, Florida; 6 NorthShore University Health System, Evanston, Illinois; 7 St George’s University Hospitals NHS Foundation Trust, London, United Kingdom; 8 Carl-Thiem Klinikum gGmbH, Cottbus, Germany; 9 Loma Linda University Medical Center, Loma Linda, California; 10 MemorialCare Heart & Vascular Institute, Fountain Valley, California; 11 Cardiovascular Associates of Mesa, Mesa, Arizona; 12 Cardiac Electrophysiology, Texas Cardiac Arrhythmia Institute, Dallas, Texas; 13 Scripps Health, La Jolla, California; 14 Mayo Clinic, Phoenix, Arizona; 15 Ascension Providence Hospital, Detroit, Michigan; 16 Sharp Memorial Hospital, San Diego, California; 17 Department of Gastroenterology, St George’s University Hospital, London, United Kingdom; 18 Department of Medicine, Division of Gastroenterology, University of Illinois at Chicago, Chicago, Illinois; 19 Advocate Aurora Christ Medical Center, Chicago, Illinois; 20 University of Texas Southwestern Medical Center, Dallas, Texas; 21 Attune Medical, Chicago, Illinois; 22 Ten15 Ventures, Westlake, Ohio; 23 Colorado College, Colorado Springs, Colorado; 24 In Silico Science & Engineering S.A.S., Medellin, Colombia; 25 Department of Electronic Engineering, Universitat Politècnica de València, Valencia, Spain; 26 Translational Medical Device Lab, School of Medicine, University of Galway, Galway, Ireland; 27 Valencian International University, Valencia, Spain

**Keywords:** Atrial fibrillation, Radiofrequency ablation, Pulmonary vein isolation, Atrioesophageal fistula, Esophageal cooling

## Abstract

Proactive esophageal cooling for the purpose of reducing the likelihood of ablation-related esophageal injury resulting from radiofrequency (RF) cardiac ablation procedures is increasingly being used and has been Food and Drug Administration cleared as a protective strategy during left atrial RF ablation for the treatment of atrial fibrillation. In this review, we examine the evidence supporting the use of proactive esophageal cooling and the potential mechanisms of action that reduce the likelihood of atrioesophageal fistula (AEF) formation. Although the pathophysiology behind AEF formation after thermal injury from RF ablation is not well studied, a robust literature on fistula formation in other conditions (eg, Crohn disease, cancer, and trauma) exists and the relationship to AEF formation is investigated in this review. Likewise, we examine the abundant data in the surgical literature on burn and thermal injury progression as well as the acute and chronic mitigating effects of cooling. We discuss the relationship of these data and maladaptive healing mechanisms to the well-recognized postablation pathophysiological effects after RF ablation. Finally, we review additional important considerations such as patient selection, clinical workflow, and implementation strategies for proactive esophageal cooling.

## Graphical abstract

**Figure:**
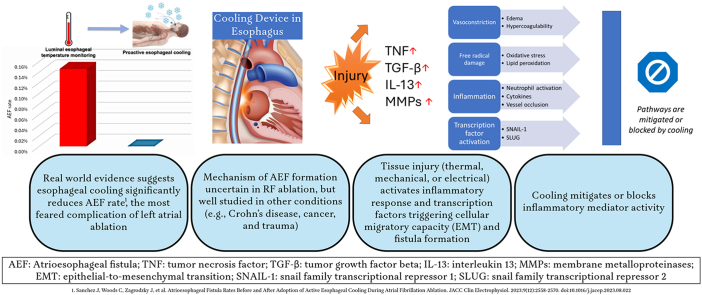

**Key Findings**

▪ Atrioesophageal fistula (AEF) is the most feared complication of left atrial ablation.
▪ Proactive esophageal cooling reduces visible esophageal injury and is associated with a significant reduction in AEF rate.
▪ Tissue injury (from thermal, mechanical, or electrical sources) triggers an inflammatory response that induces epithelial cells to become migratory, resulting in fistula formation.
▪ Cooling mitigates or blocks the activity of most of the mediators of fistula formation.

## Introduction

Radiofrequency (RF) catheter ablation is a thermally mediated method for delivering pulmonary vein isolation (PVI), a cornerstone of treatment of paroxysmal and persistent atrial fibrillation,^1^ whereby a contiguous path of transmural thermal ablation is created surrounding each of the pulmonary veins in the left atrium. Although most risks associated with PVI are manageable, the risk of collateral esophageal thermal injury, and the progression of that injury to atrioesophageal fistula (AEF) over subsequent days and weeks remains challenging to prevent, diagnose, and treat.^2^^,^^3^ Traditional strategies used to prevent esophageal injury during PVI, such as reducing RF energy duration or power and contact force, single- and multiple-point monitoring of luminal esophageal temperature (LET),^4^, ^5^, ^6^ and mechanically displacing the esophagus,^7^ have not been shown to prevent AEF. LET monitoring is the oldest and most widely used approach aimed at preventing esophageal thermal lesions; however, an increasing number of studies question the efficacy and inherent technical feasibility of this method.^4^, ^5^, ^6^^,^^8^, ^9^, ^10^, ^11^, ^12^, ^13^, ^14^, ^15^ A recent study has shown that esophageal injury can be predicted with reasonable accuracy using a postprocedure analysis of spatial and temporal LET gradients; however, this method is predictive, not preventive.^16^ Importantly, over the last 20 years, reports of AEF have not decreased, and while most other complications diminish with operator expertise, AEF does not appear to do so.^17^^,^^18^ Pulsed field ablation (PFA) is an emerging alternative cardiac ablation energy source; however, growing data from cardiac as well as oncologic applications have identified measurable and dose-dependent thermal effects with pulsed field energy.^19^, ^20^, ^21^, ^22^, ^23^, ^24^ In some cases, measured LETs during PFA have exceeded the thresholds typically used for cessation of RF delivery.^19^ In the field of oncology, where PFA has been in commercial use for over a decade, fistulas (including pancreatic, enterocutaneous, arterio-enteric, vagino-tumoral, rectovesical, and buccal) are commonly reported after PFA applications.^25^, ^26^, ^27^, ^28^, ^29^, ^30^, ^31^, ^32^, ^33^ In some reports, fistula formation occurs in as many as 10.6%–20% of patients.^25^^,^^27^

The concept of active esophageal cooling was first proposed in 2005,^34^ shortly after the first case of AEF resulting from RF catheter ablation was reported.^35^ Investigations into this concept continued for the next decade,^36^, ^37^, ^38^, ^39^, ^40^, ^41^, ^42^, ^43^, ^44^ but a practical device did not become available until 2014. Initial use of this device—the ensoETM (Attune Medical, Chicago, IL)—was in critical care, emergency medicine, and surgery for patient systemic temperature management^45^, ^46^, ^47^, ^48^, ^49^, ^50^, ^51^, ^52^, ^53^ but adoption for use during PVI has grown rapidly, with a recent analysis of >25,000 patients finding a significant reduction in AEF rate associated with its use.^54^ In September 2023, the US Food and Drug Administration (FDA) granted de novo marketing authorization for the device to reduce the likelihood of ablation-related esophageal injury resulting from RF cardiac ablation procedures.^55^ Use of this technology is now highlighted in the 2024 European Heart Rhythm Association/Heart Rhythm Society/Asia Pacific Heart Rhythm Society/Latin American Heart Rhythm Society expert consensus statement on catheter and surgical ablation of atrial fibrillation.^56^ In contrast to reactive cooling, in which cold water is administered through a nasogastric or orogastric tube into the esophagus in response to an elevated local temperature (and therefore after thermal damage has already occurred), proactive esophageal cooling involves cooling of the esophagus to 4°C before ablation lesion application.

The exact mechanism of AEF formation after RF ablation is uncertain, but the pathogenesis of fistula is generally thought to be triggered by conduction of excessive heat from a cardiac-directed RF energy application to the esophageal mucosa, often only a few millimeters away.^57^^,^^58^ Abundant data from postprocedural endoscopic studies document the prevalence and extent of transmural esophageal thermal injuries in patients after cardiac RF ablation.^59^, ^60^, ^61^, ^62^ Mucosal lesions may be just the tip of the iceberg, with periesophageal injury and the associated tissue edema and neuropathic alterations being an important component.^63^ Collateral esophageal thermal injury leads to the subsequent development of cellular changes associated with a significant inflammatory response that may lead, over the subsequent 2–12 weeks, to fistula formation.^3^^,^^64^ Although the mechanisms of AEF formation after esophageal thermal injury are difficult to study in patients, abundant literature on fistula formation exists in other conditions, particularly in Crohn disease, where up to 50% of patients develop fistulas,^65^ as well as in cancer and trauma, where fistulas occur after similar (localized) inflammatory insults. In this review, we examine the evidence behind proactive esophageal cooling and the potential mechanisms of action identified from burn, gastrointestinal, and critical care literature that may contribute to the observed reduction in AEF formation associated with proactive cooling. We discuss the relationship of these established maladaptive mechanisms to the well-recognized postablation pathophysiological effects after RF ablation. Finally, we review additional important considerations such as patient selection and usage strategies for proactive esophageal cooling.

## Acute effects

RF cardiac ablation occurs by applying RF energy to a series of specific anatomic locations, resulting in a targeted heating of cardiac tissue above a threshold where irreversible injury and necrotic cell death occur. This threshold is referred to as the *lethal isotherm*, which is the minimal tissue temperature (when exceeded for a minimum amount of time) necessary to produce permanent tissue destruction around the site of activity of the RF electrode. This tissue destruction includes physiological changes consistent with cell death, such as cellular depolarization, loss of excitability, contracture, or loss of conduction, and the threshold is estimated to range as low as 47.9°C–53.6°C and range as high as 58.1°C–64.2°C, with an inverse relationship between the absolute temperature and the time at or above the threshold.^66^, ^67^, ^68^ Importantly, there is a well-established nonlinear time component such that the greater the total heat energy deposited, the greater the injury. Energy delivery and the resulting thermal conduction toward adjacent tissues is difficult to spatially contain using only ablation tools and energy parameters. Inherently, safety concerns struggle against those of efficacy.^69^ Antenna effects may also play a role, with both RF and PFA. Conflicting data have been published in regard to this with RF ablation,^4^^,^^13^^,^^70^^,^^71^ but a recent study leveraging mathematical models suggests that there may be effects on PFA from the presence of a metal intracoronary stent near the ablation device from amplifying the electric field distortion already caused by the presence of the vessel.^72^ This spatial arrangement is different from a probe in the esophagus, and a plastic covering may reduce this effect, so more research is warranted. The heating profiles for RF and PFA are quite similar in terms of their time course and morphology, suggesting similar resistive and conductive heating profiles. This makes sense because the physical principles involved in Joule heating and tissue conduction are preserved regardless of the form of applied current. The biggest differences are the duration of energy application and the absolute magnitude of temperature change.^23^ As such, proactive esophageal cooling may also be of benefit if PFA thermal effects (particularly with increased energy deposition using newer higher-energy systems or just with greater numbers of pulses using current systems) are found to cause esophageal injury in cardiac ablation.^24^

**Figure 1:**
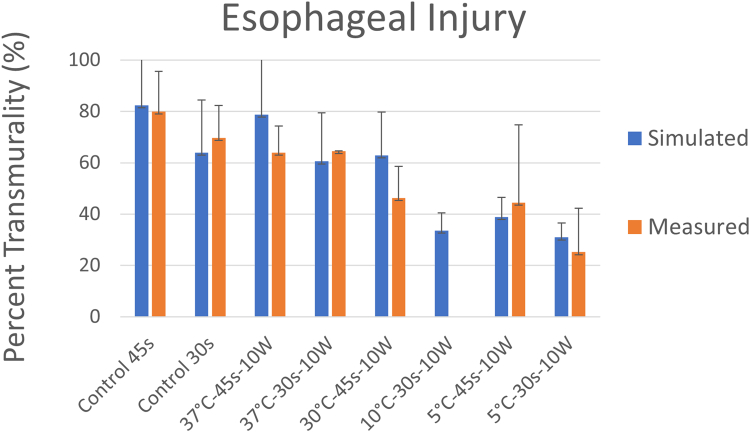
Transmurality of lesions at varying ranges of power, duration, and cooling water temperature using a dedicated active esophageal cooling device in an animal model with ablation procedures directly on the esophagus. Findings in the animal model (*orange bars*) are compared with results predicted from mathematical modeling (*blue bars*) discussed further below. Reproduced from Montoya, et al.^73^

Proactive cooling of the esophageal mucosa has the direct and immediate effect of increasing the amount of heat required in the esophagus to reach a threshold sufficient to cause clinically significant thermal damage of the esophagus. Proactive cooling directly reduces esophageal lesion transmurality, exhibiting a dose-response relationship with coolant temperature. A large animal model was developed where ablation procedures were performed under a “worst-case” condition by applying thermal energy directly on the exposed esophagus.^73^ Lesion depth measured over a range of temperatures via histopathological tissue staining showed that the transmurality of lesions decreased as circulating water temperature was decreased, with an absolute reduction in lesion depth ranging from 5% with the use of 37°C water to 45% with the use of 5°C water (Figure 1, orange bars).

**Figure 2:**
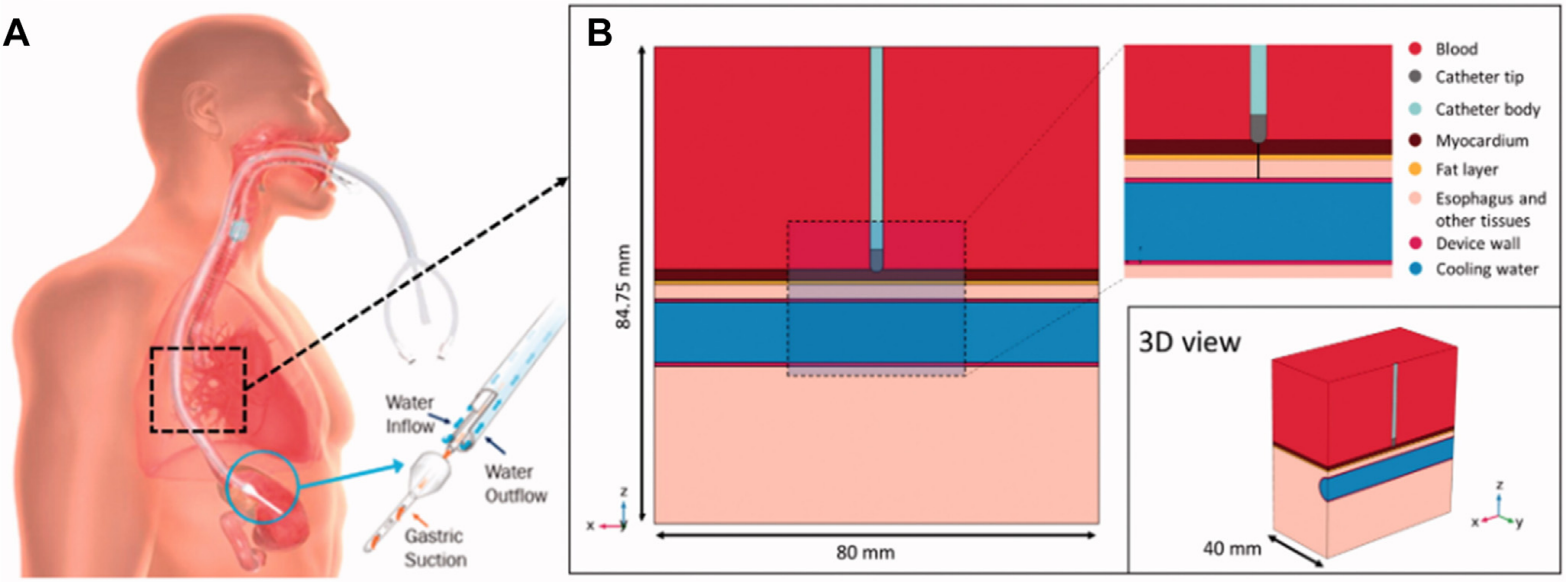
**A:** Physical situation modeled with a proactive cooling device located in the esophageal lumen. **B:** Model geometry including RF catheter, tissues near the ablation site, and proactive cooling device located in the esophageal lumen. The evaluation line (*black line*) for postprocessing is shown across the ablated tissues, from the tip of the RF catheter to the edge of the active cooling device. Reproduced from Montoya, et al.^74^ 3D = 3-dimensional; RF = radiofrequency.

A mathematical model was developed using the geometry as shown in Figure 2 to compare with the experimental data.^74^ Figure 2A shows the proactive esophageal cooling device on the left panel, and Figure 2B shows the model geometry, including all the relevant tissues and their dimensions and proximity to the ablation target. The modeled tissues include left atrial blood pool, atrial wall, epicardial fat, esophagus, and connective tissue. A cylindrical structure was assumed to be embedded into the connective tissue to model the esophageal lumen occupied by the proactive cooling device, which was modeled as a hollow silicone tube (1.2 cm diameter, 0.65 mm wall thickness) circulating cold water.

**Figure 3:**
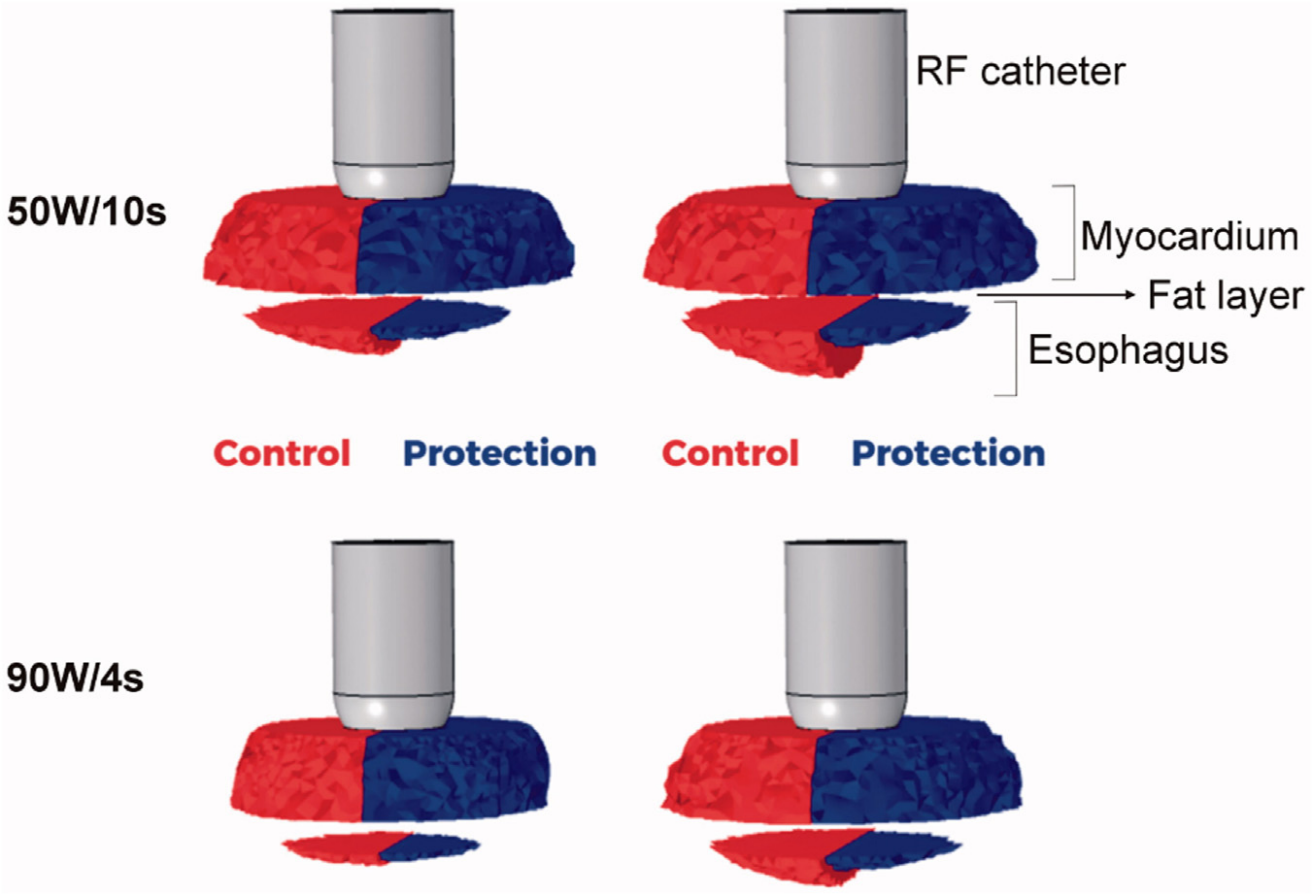
Lesion shapes for 50 W/10 s and 90 W/4 s ablation procedures, with (protection) and without (control) proactive esophageal cooling. Left-sided images show the case after the RF pulse and right-sided images show the case after 90 seconds, allowing for the effects of thermal latency. Thermal injury is not seen in the fat layer since the fraction of damage incurred by fat is lower than that of myocardial or esophageal tissue, which is a consequence of tissue parameters incorporated into the Arrhenius equation reflecting relative resistance of adipocytes to thermal insult. Reproduced from Montoya, et al.^74^ RF = radiofrequency.

Results of this model demonstrated close agreement with preclinical data (Figure 1, blue bars).^73^ Further analysis of lesion characteristics shows that proactive cooling protects against esophageal thermal insults during cardiac ablation by preventing esophageal tissues from reaching or exceeding lethal hyperthermic temperatures and also by limiting the time that esophageal tissues are hyperthermic.^74^ Notably, although cooling shows significant protective effects in the esophageal tissue adjacent to the cooling surface, the effect on atrial myocardium is negligible, with the transmurality of atrial ablation lesions remaining at 100% despite active cooling (Figure 3). Steady-state conditions show the temperature ranging from ∼12°C to 22°C across the esophagus.

The primary driver of this effect is the markedly lower heat transfer due to the limited perfusion occurring in tissues such as the visceral and parietal pericardium, serous fluid, and pericardial fat.^75^ These layers form an effective thermal insulation layer between the esophageal wall in contact with the cooling device and the atrium in contact with the RF ablation catheter. In addition, the flow of normal temperature blood through the left atrium serves as a heat source counteracting the effects of any cooling from the esophageal side. The net effect is cooling of the esophagus with little collateral cooling of the left atrium, along with heating of the atrial tissue targeted by RF ablation with little collateral warming of the surrounding esophageal tissues.^76^

Two randomized pilot studies and 1 large randomized controlled trial using esophagogastroduodenoscopy to identify esophageal lesions after ablation have been completed. Clark et al^77^ conducted the first small pilot study comparing the use of proactive esophageal cooling with a dedicated device to the use of direct instillation of cold water in response to temperature rises indicated by a single-sensor LET monitor, finding that use in the electrophysiology laboratory was feasible and that the extent of esophageal injury was less severe when using proactive cooling than with reactive manual instillation of ice cold water. Tschabrunn et al^78^ conducted the Utility of Esophageal Cooling Therapy for the Prevention of Thermal Injury During Atrial Fibrillation Ablation (E Cool-AF) trial, in which 44 patients were randomized 1:1 to receive active esophageal cooling or LET monitoring with a single-sensor probe. The investigators found a 67% reduction in severe lesions despite adjunctive posterior wall isolation being performed more frequently in patients randomized to active cooling.^78^ Leung et al^79^ conducted the Improving Oesophageal Protection During AF Ablation Randomized Controlled Trial (IMPACT), randomizing 120 patients 1:1 to active esophageal cooling or LET monitoring with a single-sensor probe. Total esophageal lesions were reduced 83%.^79^

## Delayed effects and mechanisms of fistula formation

**Figure 4:**
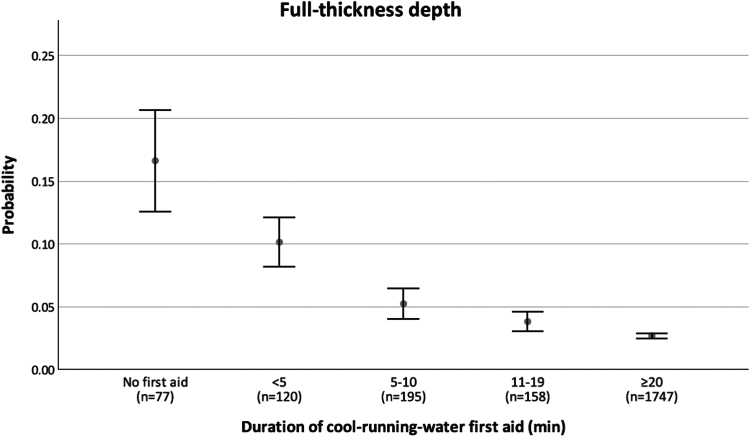
Dose-response relationship between the duration of cooling and the probability of a full thickness depth of burn. A significant inverse relation is observed between the duration of cooling and the probability of full thickness depth. Relative to burns that failed to receive any first aid cooling, those cooled with running water for lengths of ≥5 minutes had a significantly reduced probability of classification as full thickness, with progressively greater probability reductions in the 5- to 10-minute (OR 0.3; 95% CI 0.1–1.0; *P* = .04), 11- to 19-minute (OR 0.3; 95% CI 0.1–0.9; *P* = .03), and ≥20-minute (OR 0.2; 95% CI 0.1–0.4; *P* < .001) groups. Reproduced from Griffin, et al., with permission.^95^ CI = confidence interval; OR = odds ratio.

Although acute heat transfer effects are the predominant contributor to the reduction in esophageal lesion formation found clinically with esophageal cooling, the significant reduction in AEF formation seen with esophageal cooling likely involves additional downstream effects. These effects have been extensively studied and well documented in the literature of burns and thermal injuries and their healing processes.^80^, ^81^, ^82^, ^83^, ^84^, ^85^, ^86^, ^87^ As discussed above, attainment of lethal isotherm temperatures from RF energy or other hyperthermic ablation methods results in physiological changes consistent with tissue death, such as cellular depolarization, loss of excitability, contracture, or loss of conduction.^67^^,^^88^ Thermal injury subsequently progresses through known stages of burn severity, and the damage leads to development of cellular changes and, later, fistula formation.^61^^,^^64^^,^^89^ Actively cooling epidermal, dermal, or subdermal tissues after thermal injury reduces the duration of exposure to lethal isotherm temperatures,^90^ which in turn results in the formation of a less severe burn, a markedly reduced time until complete healing, and a reduction in scar area.^91^ Cooling for the treatment of thermal injury has been advocated for at least a century,^92^ and this recommendation stems from clinical experience from as far back as the time of Galen.^93^^,^^94^ Cooling has been shown to significantly reduce burn injury severity and the likelihood of progression (also referred to as *conversion*) of thermal injury in the hours to days after an initial insult. Clinically, a dose-response effect on the duration of cooling burns has been shown in a study of 2495 pediatric patients, with a threshold effect occurring at 20 minutes of cool running water (Figure 4).^95^ Even delaying the start of cooling, and cooling to only moderate (normothermic) temperature, has still shown benefit, suggesting that acute heat removal is not solely responsible for the beneficial effect of cooling a burn.^86^ Abundant evidence suggests that this effect is due to more than just dissipation of heat and instead includes alterations of cellular behavior through multiple mechanisms.^95^ These mechanisms include (1) decreasing release of lactate and histamine, (2) stabilizing thromboxane and prostaglandin levels, (3) slowing local metabolism, (4) altering membrane permeability, (5) inhibiting kallikrein activity, and (6) changing gene expression in burned tissues.^86^^,^^95^, ^96^, ^97^

Burn conversion is the process of progressive damage extending to initially uninjured tissue surrounding a burn wound followed by the dynamic process of thermal wound healing, which occurs over several days to many weeks.^98^, ^99^, ^100^ Wound progression allows an initial partial thickness injury to convert to a deep partial thickness or full thickness burn wound because of an expanding volume of tissue damage.^101^ Ischemia, inflammation, and free oxygen radicals play a role, and novel mechanisms such as autophagy have also been shown to contribute to burn progression.^99^ Thermal ablation using high-intensity focused ultrasound achieved lesion progression and further evolution to AEF in 2 of 20 animals at ∼2 weeks (10–14 days). A chronic inflammatory response, triggered by ulceration caused by high transmural esophageal temperatures, possibly exaggerated by reflux, was found. Histological analysis of tissues showed acute injury, healed (fibrotic) intermediate recovery, chronic esophagitis, and inflammatory cell infiltration to all esophageal tissue layers as well as collateral damage to the nearby vagus nerve.^88^ Preexisting esophageal vulnerability (eg, reflux-induced esophagitis) may influence esophageal lesion formation.^102^^,^^103^ Studies have found substantial impairment of the periesophageal vagal plexus after RF ablation, with damage of the plexus resulting in gastric stasis, impaired pyloric relaxation, and incompetence of the lower esophageal sphincter, thus promoting esophageal reflux.^63^

## Delayed effects and the influence of cooling

**Figure 5:**
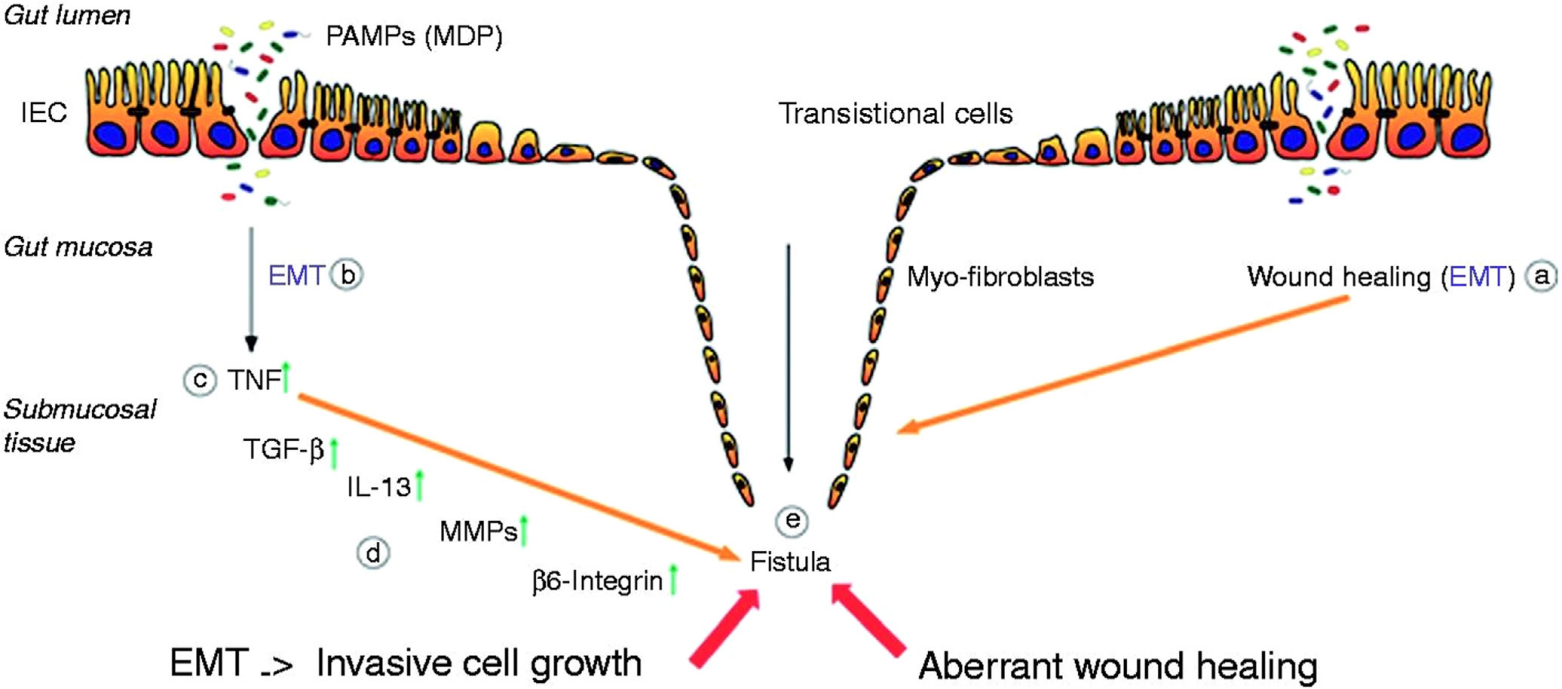
Pathogenesis of Crohn disease–associated fistulae. After an epithelial barrier defect in the gastrointestinal tract (such as would occur in the esophagus after thermal injury) several PAMPs, for example, MDP, are able to enter the gut mucosa. Both the process of wound repair (**A**) and the inflammatory response caused by PAMPs (**B**) induce the event of EMT. First, an increased expression of TNF is initiated (**C**), resulting in an upregulation of TGF-β production. This triggers the expression and secretion of IL-13 as well as of molecules associated with cell invasiveness, such as β6-integrin (**D**). The enhanced activity of MMPs, as well as the upregulation of protein expression, favors the transformation of the IECs toward the invasive myofibroblast forms, which finally results in fistula formation (**E**). Reproduced from Scharl, et al.^112^ EMT = epithelial-to- mesenchymal transition; IEC = intestinal epithelial cell; IL-13 = interleukin 13; MDP = muramyl dipeptide; MMP = membrane metalloproteinase; PAMP = pathogen-associated molecular patterns; TGF-β = tumor growth factor β; TNF = tumor necrosis factor.

Despite limited mechanistic understanding of the formation of AEF from an initial thermal insult, understanding of fistula formation in other conditions is quite advanced and fistula formation shares factors with thermal injury, inflammation, and wound repair. Fistulas occur in up to 50% of patients with Crohn disease,^65^^,^^104^ and thermal, mechanical, or electrical injury can induce a variety of fistulas, typically 1–8 weeks after initial injury. Examples include gastrocutaneous fistula,^105^ duodenocutaneous fistula,^106^ colovesicular fistula,^107^ colocutaneous fistula,^108^^,^^109^ and laryngeal fistula.^110^ Fistula formation requires a transformation in which epithelial cells develop phenotypic plasticity and lose their epithelial polarization and organization to become characteristically mesenchymal.^111^ This transition involves epithelial cells losing their characteristic properties (apicobasal polarity and epithelial-specific cell contacts) and gaining the motility of mesenchymal cells. Epithelial cells, characterized by strong intercellular junctions and cell polarity, lose their epithelial phenotype and acquire a mesenchymal differentiation featuring reduced cell-cell contacts and a fibroblast-like morphology and function, permitting these cells to become migratory, in a process referred to as epithelial-to-mesenchymal transition (EMT).^111^ Having undergone EMT, intestinal epithelial cells penetrate into deeper layers of the mucosa and the gut wall causing localized tissue damage, formation of a tubelike structure, and finally a connection to other organs or the body surface (Figure 5).^111^

The primary factors contributing to EMT include tumor necrosis factors (tumor necrosis factor α), transforming growth factors (transforming growth factor β), interleukins (interleukin 13), and matrix metalloproteinases (matrix metalloproteinase 3 [MMP-3] and MMP-9). EMT is triggered by such events as thermal injury causing an epithelial defect and is then governed by the actions of these molecular mediators, which enhance remodeling enzymes such as matrix metalloproteinases MMP-3 and MMP-9. EMT then induces gastrointestinal epithelial cells to penetrate into deeper tissue layers, form a tubelike structure, and connect to other organs, with nuclear expression of the transcription factors snail family transcriptional repressor 1 (SNAI1, or SNAIL) and snail family transcriptional repressor 2 (SNAI2, or SLUG)^113^ that are involved in the downregulation of E-cadherin.^112^ This process generally occurs over weeks, and bacterial wall components (muramyl dipeptide) may synergize with tumor necrosis factor to facilitate it.^114^ This process is also well described in oncology, where it is implicated in cancer progression.^115^ Interestingly, many of these factors are also implicated in burn wound conversion and fibrosis.^116^

Cooling has been shown to mitigate the activity of many soluble factors, chemokines, enzymes, and mediators that are activated by tissue heating and are involved in inflammatory, fibrotic, and fistula remodeling processes (Table 1).^86^^,^^117^, ^118^, ^119^ The pleiotropic effects of cooling that serve as protective mechanisms against inflammation, oxidative stress, apoptosis, and excitotoxicity have also been leveraged for clinical benefit in numerous areas of medicine, including neonatology, ophthalmology, and critical care.^120^, ^121^, ^122^

**Table 1:** Molecular mediators are implicated in the pathophysiology of burn injury progression, fibrosis, and fistula development

Soluble factors, mediators, circulating chemokines, remodeling enzymes	Action in burns	Action in fistula formation	Action in fibrosis	Temperature effects	Effect of cooling on activity or expression
TNF- α	Proinflammatory cytokine	Triggers EMT, onset and progression of fistula formation	Induces apoptosis of fibrotic progenitors	Cooling significantly reduces activity	
TGF-β	Worsens scar formation	Triggers EMT, onset and progression of fistula formation	Induces fibrosis	Cooling reduces mRNA expression	
Angiotensin II	Possible synergistic signal to TGF-β in burn scarring	Unclear actions	Expression of genes related to fibrosis	Lowers body temperature when administered systemically	
IL-1	Proinflammatory cytokine (inhibited by IL-1ra)	Inhibition alleviates severe fistulae in hidradenitis suppurativa	Profibrotic cytokine induces apoptosis of fibrotic progenitors	Downregulated or unchanged with cooling	
IL-4	Anti-inflammatory cytokine	Implicated in oronasal fistula formation	Facilitates muscle regeneration	Expression levels of IL-4 anti-inflammatory cytokines increased	
IL-6	Proinflammatory cytokine associated with mortality	Induced by TNF-α, increases permeability of the endothelial layer	Triggers cardiac fibrogenic signaling cascade	Reduces IL-6 expression	
IL-7	Proinflammatory cytokine	Unclear actions	May inhibit high glucose-induced renal proximal tubular fibrosis	Uncertain	
IL-8	Enhances neutrophil transmigration; proinflammatory cytokine associated with ARDS	Putative role in the pathogenesis of cryptoglandular anal fistula	Dominates the inflammatory profile in cystic fibrosis	Higher levels may determine severity hypoxic ischemia	
IL-10	Anti-inflammatory cytokine, associated with burn mortality	Impaired IL-10 signaling implicated in inflammatory bowel fistulas	Profibrotic cytokine	Elevations delayed with hypothermia	
IL-12	Proinflammatory cytokine stimulates the production of TNF-α	Elevated levels linked to enterocutaneous fistulas	Induces apoptosis of fibrotic progenitors	Reduced expression with hypothermia	
IL-13	Anti-inflammatory cytokine induces metaplasia	Triggers EMT, onset and progression of fistula formation	Effects muscle regeneration by resident mesenchymal progenitor cells	Expression levels increased with hypothermia	
IL-17	Proinflammatory cytokine increased during burn injuries	Proinflammatory mediator; key role in fistula formation in hidradenitis suppurativa	Mediator in foreign body response and fibrosis	Gene expression levels significantly downregulated with local cryotherapy	
Matrix metalloproteinases (MMPs): MMP-1, MMP-3, MMP-9	Upregulated in vascular inflammation	Activated by EMT, causing further tissue damage and inflammation	Associated with fibrotic processes underlying right ventricular remodeling	Downregulatory effects on expression	
Histamine	Increases wound edema, microvascular permeability	Unclear actions	Unclear actions	Decreases or prevents histamine release	
Reactive oxygen species	Induce burn progression, edema formation, and microvascular permeability	Implicated in enterocutaneous fistula development	Associated with severity of cystic fibrosis	Reduced with hypothermia	

Both fibrosis and epithelial-to-mesenchymal transition (EMT) are drivers of fistula development after an initial thermal injury, and the soluble mediators of these processes are also implicated in burn wound conversion, which further facilitates the progression of thermal injury. The activity of many proinflammatory markers is inhibited by cooling.

ARDS = acute respiratory distress syndrome; IL-1 through IL-17 = interleukin 1 through 17; MMP = matrix metalloproteinase; TGF-β = tumor growth factor beta; TNF-α = tumor necrosis factor alpha.

Cooling induces the upregulation of skin-protective genes and downregulation of detrimental tissue remodeling genes, and this can be seen even when cooling is delayed by 2 hours.^85^ Gene alterations result from burn injury, and the number of permutations that can occur are extensive, involving as many as 2286 genes.^97^ A large number of inflammatory markers have been shown to be inhibited by cooling.^80^^,^^86^^,^^93^^,^^117^, ^118^, ^119^^,^^123^, ^124^, ^125^, ^126^, ^127^, ^128^, ^129^ Downregulatory effects are seen on the expression of MMP-9 mRNA and upregulating effects on the expression of chemokine (C-X-C motif) ligand 13 (CXCL13), lipopolysaccharide binding protein, and chemokine (C-C motif) ligand 6 (CCL6) and chemokine (C-C motif) ligand 24 (CCL24). These molecules have important functions in B-cell maturation, reduction in endotoxin load and improved bacterial opsonization, keratinocyte proliferation, and collagen synthesis and deposition by fibroblasts.^86^ Increased vascular permeability is another proposed contributor to thermal injury progression. Reports from the 1940s showed that the enhanced vascular permeability resulting from burns could be reduced with local cooling.^84^^,^^130^^,^^131^ Improved burn healing or reduced wound progression has been shown via inhibition of the increase in permeability of capillaries in the burned area, limiting edema formation.^81^, ^82^, ^83^ Edema formation is inhibited even if cooling is undertaken up to 30 minutes after burn injury,^132^ while improved healing is seen with shorter delays to cooling.^80^ Biopsies show an earlier and more rapid rate of growth of epithelial cells, less tissue necrosis, and less final fibrosis in cooled tissues.^93^ The fact that even delayed cooling (when tissue temperatures have long since returned to normal) results in improved outcomes underscores that favorable effects of cooling are not explained by heat removal alone.^80^^,^^86^

## Patient selection, clinical considerations, and implementation strategies

**Figure 6:**
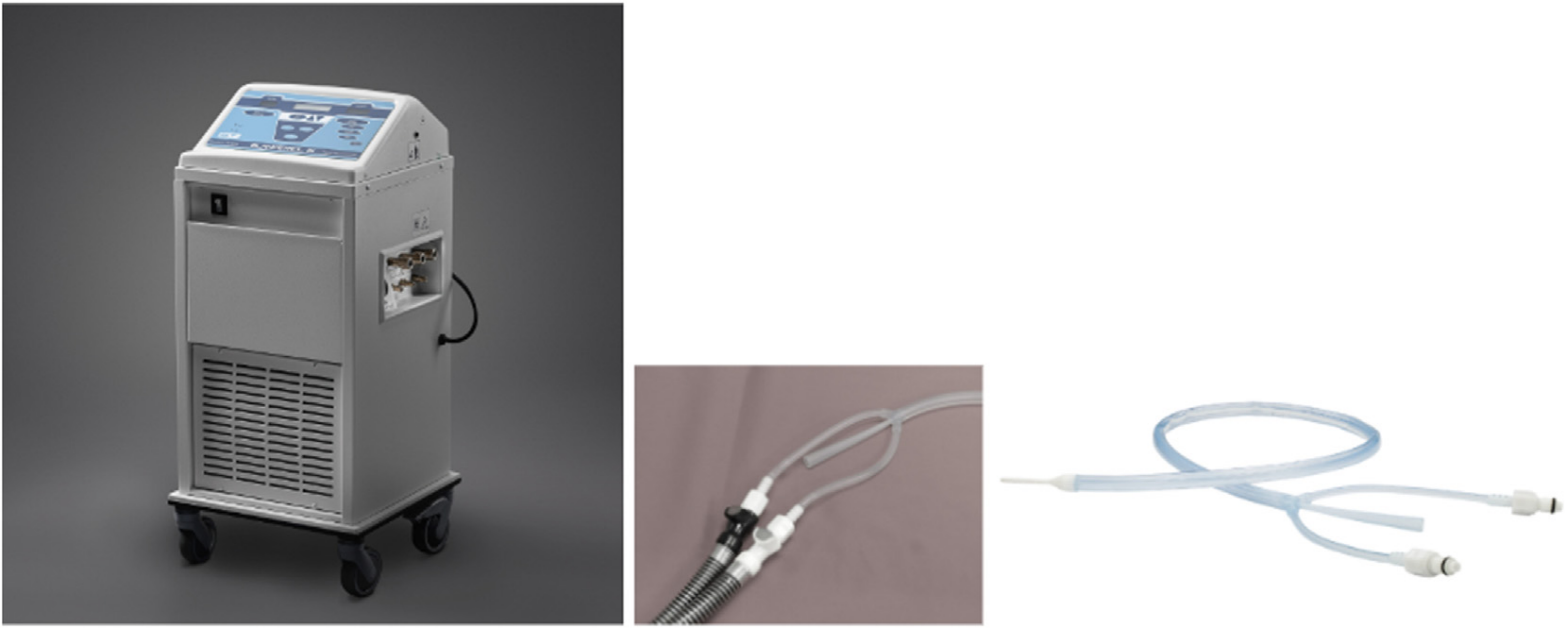
Example heat exchanger, connector tubing, and esophageal cooling device.

Proactive esophageal cooling for the purpose of reducing the likelihood of ablation-related esophageal injury resulting from RF cardiac ablation procedures is the only FDA-cleared protective strategy currently commercially available. PFA has potential to replace RF ablation for many cardiac ablation procedures, but while PFA was initially believed to be inherently safe because of purported cardiac tissue selectivity and a nonthermal mechanism of action, the clinical evidence surrounding PFA is still emerging and unexpected risks are still being identified.^19^, ^20^, ^21^, ^22^, ^23^^,^^133^, ^134^, ^135^, ^136^, ^137^, ^138^, ^139^, ^140^, ^141^, ^142^, ^143^, ^144^ Proactive esophageal cooling has robust clinical evidence documenting a reduction in esophageal injury and AEF formation after RF ablation.^54^^,^^79^ The use of proactive esophageal cooling has also shown improved workflow as assessed by reduced procedure time,^145^ reduced fluoroscopy requirements,^146^ and improved long-term efficacy, presumably because of the ability to deliver RF energy in the intended range while applying contiguous lesions.^147^, ^148^, ^149^ An improvement in the continuity of lesions, quantified by the continuity index defined in the TactiCath® Prospective Effectiveness Pilot Study (EFFICAS-II), is associated with improved long-term freedom from arrhythmia.^150^, ^151^, ^152^ The FDA-cleared esophageal cooling device is a closed-loop, multilumen medical grade silicone tube placed into the esophagus in a manner analogous to a standard orogastric tube. The device is connected to an external heat exchanger, which circulates chilled water to provide heat transfer (Figure 6). The connector tubing provides flexibility in placement of the heat exchanger, and a central lumen in the device allows gastric access for suctioning and decompression.

### Patient selection

Adopters of proactive esophageal cooling generally use cooling for any patient undergoing RF ablation that may include the posterior wall of the left atrium, including standard PVI procedures and ablation of left atrial tachycardias. Since esophageal location can vary across the left atrium and can move during the procedure, a priori determination of esophageal risk remains challenging.^153^ Some operators will also include patients in whom ablation involving the coronary sinus (CS) is likely, as ablation in the CS is an independent risk factor for esophageal lesions because of the epicardial location close to the posterior left atrial wall. No formal contraindications for esophageal cooling exist, but the device instructions warn against use in patients with known esophageal deformity or evidence of esophageal trauma or in patients known to have ingested acidic or caustic poisons within the prior 24 hours.

### Implementation

The device is generally placed at the time of anesthesia induction. Centers in the United States typically use general anesthesia, whereas many in Europe use conscious or deep sedation. In cases using esophageal cooling with sedation, atropine may be given to reduce salivation. If transesophageal echocardiography (TEE) is planned, then TEE is performed and the TEE probe removed before placing the cooling device. Placement after the creation of a 3-dimensional (3D) map should be avoided because, as with any esophageal manipulation, the geometry of the posterior wall may be changed, especially in patients with low body mass index. Since anesthesia induction precedes the mapping and ablation procedure, this is not a common issue and, once placed, there is generally no need for further adjustment or manipulation of the cooling device during the procedure. Cooling can begin immediately, but should begin at least several minutes before beginning ablation, with the water temperature set at 4°C. No data exist to specify a duration for cooling after the completion of ablation, although data extrapolated from the burn literature suggest additional benefit of cooling for up to 20 minutes after the final ablation on the posterior wall.^95^^,^^154^

### Efficiency considerations

**Figure 7:**
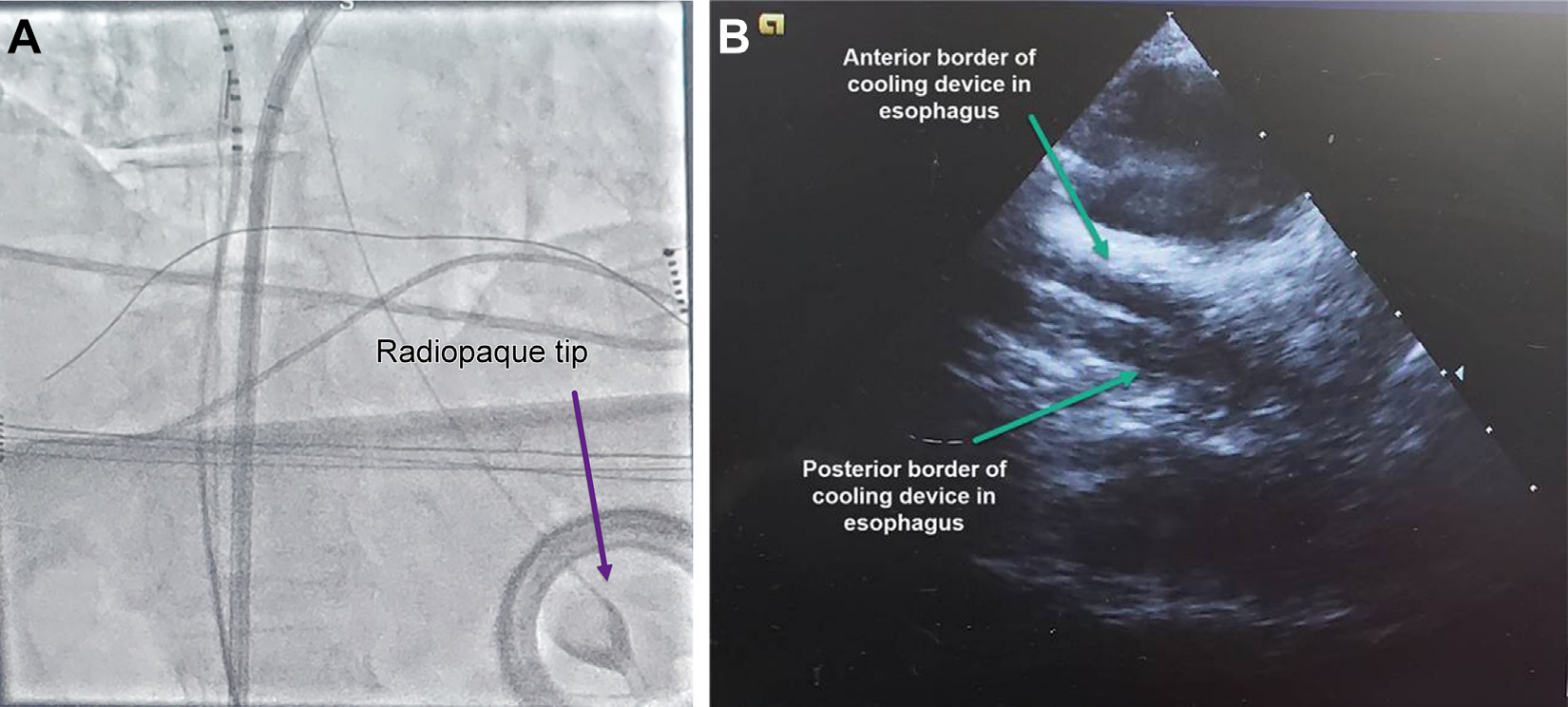
**A:** Visualization of the proactive esophageal cooling device (ensoETM, Attune Medical, Chicago, IL) on fluoroscopy, showing the radiopaque tip below the diaphragm. **B:** Visualization of the proactive esophageal cooling device (ensoETM, Attune Medical) on intracardiac echocardiography, showing anterior and posterior borders of the device in the esophagus.

In most laboratories, the anesthesiologist or certified registered nurse anesthetist (CRNA) will place the cooling device but further efficiency has been found with training of laboratory staff (such as the electrophysiology nurse) to place the device, particularly when multiple anesthesia personnel staff the electrophysiology laboratory. The device requires lubricating before placement, and placement typically takes ≤3 minutes (similar to a standard orogastric tube).^155^ Gentle torsion of the device can ease insertion, and observing for kinking in the posterior oropharynx is recommended. Occluding the outflow of the device to increase stiffness from the increase in water pressure can further enhance the ease of placement. Fluoroscopy or intracardiac echocardiography (ICE) can be used to determine proper placement and ensure that the cooling device contacts the entire left atrial posterior wall. Additional radiopacity is provided by some operators via instillation of 5 mL of oral contrast medium (eg, diatrizoate [Gastrografin]), or placement of a guidewire, into the central (gastric) lumen of the device. In cases using no fluoroscopy and no ICE, visualization of the cooling device can be obtained on the 3D electroanatomic map by passing an SL-1 (0.032 in, 150 cm length) guidewire (Abbott, Chicago, IL) through the central lumen of the cooling device.^156^ The guidewire is then pinned via a pin block to the cardiac mapping system (EnSite, Abbott, St. Paul, MN), and a unipolar configuration is used to visualize the guidewire tip on the map passing below the CS. The minimal depth of the device should be such that the radiopaque tip (Figure 7A) is just below the diaphragm, but placing it 8 - 12 cm further below the diaphragm affords additional safety against inadvertent retraction. There is no depth limit, as the design is intended for placement as far as the pyloric antrum. Some operators use the diagnostic CS catheter as a landmark, confirming tip placement below a properly placed CS catheter. On ICE, the device can be seen clearly after clocking the ICE catheter posteriorly to visualize the esophagus (Figure 7B).

The presence of active water flow through the device can be confirmed by visualizing the on-screen, side-mounted, or in-line flow indicators. Securement of the device should be ensured, with the connecting hoses placed and secured in such a manner (such as under the arm board) to prevent inadvertent tension on the device resulting in dislodgment. Avoiding contact of cool surfaces with patient skin can improve patient comfort. Patient temperature is typically measured via axillary placement of a temperature probe, but it is important to remember that this temperature is commonly up to 2°C colder than the core temperature.^157^ As such, adding this difference is necessary to obtain the actual core temperature when using an axillary measurement. Because induction of general anesthesia typically reduces patient temperature by 0.5°C–1.5°C,^157^ forced-air warming is often used for patients in the electrophysiology laboratory. The addition of cooling for the duration of left atrial ablation procedures does not typically result in significant decreases in patient temperature, but for longer procedures, or for patients with low body mass index, ensuring properly placed forced-air warming blankets or providing head covering may be advantageous. Recent randomized controlled trial data have found no detriment to cooler surgical patient temperatures than traditionally targeted,^158^ and consequently, operative patient temperature guidelines are expected to be revised. Some operators will annotate the 3D map with the esophageal cooling device location. Optimal practice is to reconfirm proper cooling device location before ablation application near the posterior wall. Cooling can be continued after RF applications to further reduce inflammation while the patient is prepared for awakening, sheath removal, and extubation. Some operators prioritize posterior wall lesions early in the case to shorten duration of cooling at the conclusion of the case, which can further improve workflow efficiency. Once ready for removal, attaching suction to the device gastric lumen may help to evacuate any residual gastric contents.

### Troubleshooting

Difficulties in placement can generally be addressed by optimal positioning of the patient and placing a generous amount of lubrication on the distal 15–20 cm of the device. Extending the neck to straighten the oropharyngeal axis will reduce the angle of curvature required to pass through on initial entry into the esophagus. A jaw thrust can further open this passage. External flow obstruction (such as accidental kinking of the connector hose anywhere along the path or the device at the head of the bed) will trigger an audible alarm, which should be investigated immediately. Likewise, inadequate water levels in the heat exchanger will trigger audible alarms; however, a low water level alarm during use should prompt investigation to ensure the absence of any device leak. Water levels should be checked routinely, and the heat exchanger should be filled with sterile water at regular intervals.

## Conclusion

Esophageal thermal injury caused by collateral spread of ablation energy intended for the left atrium may trigger an evolving cellular transition that can progress to AEF formation over the course of days to weeks. Temperature monitoring and esophageal deflection methods have not demonstrated a reduction in AEF formation, and it is too early to be certain that current or future PFA systems will eliminate the risk of AEFs; however, in multiple clinical trials, proactive esophageal cooling has shown significant reduction in esophageal injuries and AEF after RF ablation. Although cooling shows significant protective effects in the esophageal tissue adjacent to the cooling surface, the effect on atrial myocardium and the resulting effectiveness of thermal ablation appears negligible, with the transmurality of atrial ablation lesions remaining at 100% despite active esophageal cooling. Based on the established mechanisms known from related conditions, the primary contributors to AEF formation appear to be proinflammatory mediators and matrix remodeling enzymes. The activity of a majority of these mediators is triggered by thermal injury, whereas most of these mediators are inhibited by a reduction in peak temperature, a shortening of the duration of elevated temperature, and the application of cooling. As long as thermal ablation of the left atrium is a treatment modality, active esophageal cooling should be considered as part of the standard procedural workflow as a safety-enhancing strategy that targets the fundamental drivers of esophageal thermal injuries and their downstream sequelae while also leading to improved procedural efficacy and efficiency.
